# Epidemiological African day for evaluation of patients at risk of venous thrombosis in acute hospital care settings

**DOI:** 10.5830/CVJA-2014-025

**Published:** 2014

**Authors:** Samuel Kingue, Limbole Bakilo, Richard Mvuala, Jacqueline Ze Minkande, Inoussa Fifen, Yash Pal Gureja, Henri Jean Claude Razafimahandry, Njideka Okubadejo, DA Oke, Alexandre Manga, Tovohery Rajaonera, Nwadinigwe Cajetan, Emmanuel Pay Pay, Nirina Rabearivony

**Affiliations:** Department of Cardiology, General Hospital, Yaoundé, Cameroon; Department of Internal Medicine, Clinic Ngaliema, Kinshasa, DRC; Department of Internal Medicine, Clinic Ngaliema, Kinshasa, DRC; Department of Anaesthesiology and Reanimation, Hospital Gynéco obstetrique et Pédiatrique, Yaoundé, Cameroon; SA NOFI, North-East Africa, Lagos, Nigeria; Orthopaedic Department, Princess Marina Hospital, Gaborone, Botswana; Orthopaedist, Antananarivo, Madagascar; Department of Neurology, Lagos University Teaching Hospital, Lagos, Nigeria; Department of Cardiology, Lagos University Teaching Hospital, Lagos, Nigeria; Orthopaedic Department, Hopital du Caisse nationale de prévoyance Sociale, Yaoundé Cameroon; Department of Anaesthesiology and Reanimation, Mother and Child Hospital, Antananarivo, Madagascar; National Orthopaedic Hospital, Enugu, Nigeria; Orthopaedic Department, Clinic Ngaliema, Kinshasa, DRC; Department of Cardiology, Mother and Child Hospital, Antananarivo, Madagascar

**Keywords:** thromboprophylaxis, venous thromboembolism, ENDORSE-Africa

## Abstract

**Introduction:**

This study aimed to identify patients at risk for venous thromboembolism (VTE) among all patients hospitalised, and to determine the proportion of at-risk hospital patients who received effective types of VTE prophylaxis in sub-Saharan Africa (SSA).

**Methods:**

A multinational, observational, cross-sectional survey was carried out on 1 583 at-risk patients throughout five SSA countries.

**Results:**

The prevalence of VTE risk was 50.4% overall, 62.3% in medical and 43.8% in surgical patients. The proportion of at-risk patients receiving prophylaxis was 51.5% overall, 36.2% in medical and 64% in surgical patients. Low-molecular weight heparin was the most frequently used prophylactic method in 40.2% overall, 23.1% in medical and 49.9% in surgical patients.

**Discussion:**

This study showed a high prevalence of VTE risk among hospitalised patients and that less than half of all at-risk patients received an American College of Clinical Pharmacy-recommended method of prophylaxis.

**Conclusion:**

Recommended VTE prophylaxis is underused in SSA.

## Abstract

Acute venous thromboembolism (VTE) is a complication in patients hospitalised for a wide variety of acute medical and surgical conditions.^1^,^2^ In developed countries, VTE is the most common preventable cause of death among hospitalised patients. Over the last 30 years, extensive research has demonstrated a high risk of VTE in patients who undergo major surgery or experience severe trauma. Patients hospitalised for acute medical illness have approximately the same level of VTE risk as patients who undergo major general surgery.^3^-^5^

The benefits of VTE prophylaxis are similar for both medical and moderate-risk surgical patients.^6^,^7^ VTE prophylaxis is substantially underused. There is great variation in the use of prophylaxis between countries. Even when prophylaxis is used, it may be used sub-optimally.^8^-^10^ Although some surveys and studies suggest that physicians have begun to recognise VTE as a serious health problem and use prophylaxis for at least some high-risk patients, a number of recent studies demonstrate that VTE prophylaxis remains underutilised.^11^-^20^

## Study rationale and objectives

A previous study, ENDORSE, was conducted in 32 countries worldwide, except in sub-Saharan Africa. The results of this study confirmed the high prevalence of patients at risk for VTE; about 52%, among medical and surgical hospital patients. Despite the high prevalence of VTE risk factors, only 58.5% of surgical patients and 39.5% of medical patients received thromboprophylaxis.^21^ ENDORSE-Africa aimed to collect specific African data on thromboprophylaxis and to structure the doctors’ and other healthcare stakeholders’ awareness on the challenges of VTE prophylaxis.

The primary objectives of this survey were (1) to identify patients at risk for VTE among all patients hospitalised in representative hospitals throughout some African countries, and (2) to determine the proportion of at-risk hospital patients who receive effective types of VTE prophylaxis.

The secondary objectives were: (1) to define the overall rate of appropriate prophylaxis in medical versus surgical populations, (2) to define the global rate of patients at risk by type of acute illness (medical and/or surgical), (3) to describe the factors driving the prophylaxis decisions; (4) to carry out all preceding analyses in each country.

## Methods

The study was a multinational, observational, cross-sectional survey on prevalence of VTE risk in hospital patients. The survey aimed at collecting information on the prevalence of VTE risk in hospital patients in selected sub-Saharan African countries. No intervention or modification of standard care was carried out.

Subjects were evaluated during a single audit visit. However, the information regarding VTE risk, prophylaxis and bleeding risk were recorded retrospectively to reflect the conditions present on admission or that developed within the first 14 days following hospital admission, regardless of the overall length of stay of a given patient. The focus on the first 14 days following hospital admission reflected the evidence base for the survey, that is, randomised clinical trials of VTE prophylaxis in a wide variety of at-risk hospitalised patients.

In published trials, patients at risk were identified and VTE prophylaxis was begun at or soon after hospital admission. This survey therefore focused on the first 14 days following hospital admission for an acute medical illness or major surgery. No follow-up data were collected.

In the medical group, patients 40 years of age or older who were admitted for treatment of a serious medical illness and/or were admitted for a major traumatic event that required no major operation (including closed head injury) were included in the study. In the surgical group, to be included in the surgical arm of the study, patients had to be 18 years or older and to have undergone a major surgical operation requiring general or epidural anesthesia lasting at least 45 minutes.

Patients were excluded from the study on one criterion; exclusion by type of illness. Patients admitted to or cared for in a hospital ward, floor or care unit dedicated to psychiatric, paediatric, palliative care, maternity, ear, nose and throat, burns, dermatological/ophthalmological, alcohol/drug treatment were excluded from the survey. Patients in bed on the day of this survey in eligible wards (clinical units) were reviewed individually for eligibility to participate in this survey.

This survey was conducted in accordance with the principles laid down by the 18th World Medical Assembly (Helsinki, 1964) and all subsequent amendments and guidelines for good epidemiology practice. In addition, each participating country obtained all necessary regulatory and ethics approvals in accordance with local regulations.

## Data collection

The survey was conducted in hospitals in one or two big cities of each participating country. The participating hospitals were selected from a complete list of all hospitals in each city.

Hospitals dedicated exclusively to psychiatric care, care of children, rehabilitation/hospice care, or scheduled for only major operations were excluded. Hospitals with more than 50 beds that admit patients for treatment of acute medical illnesses or exacerbation of chronic disease, and schedule routine major surgical procedures were included in this survey. Data were collected using the following tools and methods.

A hospital information questionnaire was completed by the lead investigator at each participating hospital. This was used to collect the following information on each participating hospital: hospital address, type of hospital (general/speciality), number of hospital beds, number of ICU beds, services available at the hospital (e.g. orthopedic surgery, ICU, emergency room), name and contact information of the ENDORSE investigator at the hospital, types of VTE prophylaxis that are available for use (formulary coverage), annual number of patient discharges, hospital-wide standards for VTE prophylaxis, types of patient wards (care units) that were not eligible for inclusion in ENDORSE, and total number of patients in units not eligible for this audit.

A ward questionnaire was completed by the investigator or his/her co-workers on the day of the ENDORSE audit, at each participating hospital. The ward information questionnaire was used to collect the following information on each participating hospital ward: total number of patient beds in the ward, ward standards for VTE prophylaxis, number of beds occupied on the day of the ENDORSE audit, and for ward patients who were not eligible for enrollment in ENDORSE, the reason for ineligibility.

A two-page patient case report form (CRF), was completed for each patient who was hospitalised in a qualified ward and who was otherwise eligible for enrollment. For eligible patients whose medical charts were available, the following information was collected and entered in the CRF: demographic information (age, gender), weight and height, type of major surgical procedure performed or major medical illness present on admission or during the first 14 days of hospitalisation, day of admission (as day 1 up to day 14) on which the surgical procedure/major medical illness was recorded in the chart (diagnosed), additional risk factors for VTE present during the first 14 days of hospitalisation, risk factors for bleeding present during the first 14 days of hospitalisation, day of hospitalisation on which the VTE risk factor/bleeding risk factor was first noted in the chart (first to 14th), type of VTE prophylaxis used and the day of hospitalisation on which it was initiated /terminated, presence/absence of the therapeutic anticoagulation, and the day of hospitalisation on which it was initiated/terminated. The two-page case report form was collected and sent to data management for analysis.

## Evaluation criteria

The main evaluation criteria were the proportion of patients at risk of VTE among patients hospitalised in medicine and surgery, and the proportion of these patients at risk who received proper thromboprophylaxis during the first 14 days of admission. The secondary criteria were the proportion of at-risk patients by type (medical versus surgical), the proportion of at-risk patients properly prophylaxed by type (medical versus surgical), and the factors determining the decision of the type of prophylaxis given.

## Statistical analysis

The primary objective of ENDORSE was to assess the prevalence of VTE risk in hospitalised patients. In order to assess the true prevalence of VTE risk at 50%, confidence interval of 95% with a precision of ± 3%, a minimum of 1 068 patients were needed. Based on the assumption of 10% missing data, a minimum of 1 186 patients should be included. The study aimed at including 1 200 patients, with the following split by country: Madagascar: 400; Nigeria: 400; Cameroon: 400; Democratic Republic of Congo: 300; Namibia: 100 patients.

Population characteristics (including demographics, medical history, nature, duration and severity of the disease, co-morbidities, current treatment or no treatment) were summarised into numbers of non-missing data, mean, standard deviation, minimum, maximum, median, 95% confidence interval (CI) of the mean for quantitative variables, and number and percentage with 95% CI of the population for categorical data.

The prevalence of VTE risk defined as risk factors meeting the minimum criteria established by the American College of Chest Physicians (version 2004) and any other valid guidelines available at the time of data collection were provided for both the medical and surgical population, with 95% confidence interval by country. Analysis was carried out based on populations where level of data was satisfactory. The distribution of patients by range of VTE risk factors and the distribution of patients by type of VTE prophylaxis were provided. The prevalence of prophylaxis for VTE is provided by country and globally.

## Results

Between June 2009 and December 2010, eligible patients were enrolled from 14 hospitals across five sub-Saharan African countries (Madagascar, Democratic Republic of Congo, Cameroon, Botswana and Nigeria). A total of 1 623 patients were surveyed; 567 were medical patients, 1 016 were surgical patients and 27 were unclassified due to incomplete information. Twelve subjects violated the protocol while one subject was below the required age for inclusion. Therefore, the overall number of subjects assessed was 1 583.

Reasons for hospital admission and general characteristics of the patients are shown in Tables 1, 2 and 3. The median age of the surgical patients was 45 (IQR 33–59) years, median body mass index (BMI) was 24.2 (IQR 20.9–28.9) kg/m^2^, and 290 51.2%) were female. The median age of the medical patients was 60 (IQR 51–72) years, median BMI was 23.5 (IQR 20.5–26.7) kg/m^2^, and 521 (51.3%) were male. Due to missing values, the BMI could only be calculated for 374 surgical and 196 medical patients. The median length of hospital stay up to the survey date was five days in the overall study population.

**Table 1:** Characteristics and reasons for admission of assessable surgical patients

*Reason for hospitalisation*	*Met VTE risk criteria (n = 445) (%)*	*Did not meet VTE risk criteria (n = 571) (%)*
Age, mean ± SD	45.15 ± 16.59	48.95 ± 17.24
Gender, M (%)	153 (34.4)	368 (64.4)
F (%)	292 (65.6)	203 (35.6)
Hip replacement	49 (11.0)	2 (0.4)
Knee replacement	7 (1.6)	2 (0.4)
Hip fracture	19 (4.3)	1 (0.2)
Curative arthroscopy	–	1 (0.2)
Other orthopaedic trauma	2 (0.5)	219 (34.8)
Colon/small bowel surgery	47 (10.6)	2 (0.4)
Rectosigmoid surgery	2 (0.5)	2 (0.4)
Gastric surgery	10 (2.3)	–
Hepatobiliary surgery	1 (0.2)	5 (0.9)
Urological surgery	31 (7.0)	1 (0.2)
Vascular surgery	1 (0.2)	7 (1.2)
Thoracic surgery	1 (0.2)	4 (0.7)
Gynaecological surgery	169 (38.0)	1 (0.2)
Other surgery	16 (3.6)	107 (18.6)
Admitted with major trauma but surgery not done	–	–

**Table 2:** Assessable surgical patients presenting with more than one surgery

*Reason for hospitalisation*	*Met VTE risk criteria (n = 445) (%)*	*Did not meet VTE risk criteria (n = 571) (%)*
Age, mean ± SD	45.15 ± 16.59	48.95 ± 17.24
Gender, M (%)	153 (34.4)	368 (64.4)
F (%)	292 (65.6)	203 (35.6)
Urological and other surgery	1 (0.2)	1 (0.2)
Hip fracture and other surgery	–	1 (0.2)
Thoracic and other surgery	1 (0.2)	–
Other orthopaedic trauma and other surgery	–	2 (0.4)
Gastric and hepatobiliary surgery	1 (0.2)	–
Gynaecological and colon/small bowel surgery	–	1 (0.2)
Hip and knee replacement surgery	–	1 (0.2)
Colon/small bowel and thoracic surgery	–	1 (0.2)
Hip replacement and hip fracture	–	1 (0.2)

**Table 3:** Characteristics and reasons for admission of assessable medical patients

*Reason for hospitalisation*	*Met VTE risk criteria (n = 353) (%)*	*Did not meet VTE risk criteria (n = 214) (%)*
Age, mean ± SD	63.0 ± 12.8	59.0 ± 12.2
Gender, M (%)	103 (48.1)	174 (49.3)
F (%)	111 (51.9)	179 (50.7)
Acute heart failure	179 (50.7)	–
Ischaemic stroke	113 (32.0)	–
Haemorrhagic stroke	32 (9.1)	–
Other cardiovascular disease	277 (78.5)	143 (66.8)
Haematological diseases	50 (14.2)	38 (17.7)
Malignancy (active)	42 (11.9)	–
Acute non-infectious respiratory disease	42 (11.9)	–
Pulmonary infection	136 (38.5)	–
Infection (non-respiratory)	49 (13.9)	86 (40.2)
Haematological or inflammatory	18 (5.1)	23 (10.7)
Neurological disease	21 (5.9)	38 (17.7)
Renal disease	59 (16.7)	67 (31.3)
Endocrine/metabolic disease	100 (28.3)	101 (47.2)
Gastrointestinal/hepatobiliary disease	46 (13.0)	42 (19.6)
Other medical conditions	37 (10.5)	47 (22.0)

Of the 1 583 enrolled patients, 798 (50.4%) were deemed to be at risk for VTE; 445 (43.8%) of the 1 016 surgical patients were at risk, as were 353 (62.3%) of the 567 medical patients. There were 88 surgical patients who were at medical risk for VTE. Cardiovascular diseases, endocrine/metabolic diseases and active malignancy were the commonest conditions and/or reasons for admission among surgical patients at risk of VTE, while cardiovascular diseases, endocrine/metabolic diseases, and infections (including pulmonary) were the commonest among medical patients.

Of the surgical patients, 454 (44.5%) were admitted with major trauma, while only three (0.5%) of the medical patients had major trauma on admission. Among the surgical patients admitted with a major trauma, 46 (4.5%) had hip fractures, 27 (2.7%) had head injuries while the rest had mixed types of trauma or other forms of trauma. Orthotrauma and gynaecological conditions were the commonest causes/reasons for post-admission operations among the surgical patients with 219 (21.6%) and 169 (16.6%) undergoing the respective operations. Forty patients (5.7%) had operations due to cancer.

Sixty-two (6.1%) surgical patients had operations performed prior to admission, while 437 (43%) had operations performed within the first two days of admission. A total of 271 (38.5%) of the surgical patients had emergency surgery performed. Risk factors for VTE that were present before admission are shown in Table 3.

Of the 798 patients at risk of VTE prior to admission, 471 (59.0 %) were female; the median age was 54.07 (IQR 39–66) years. In the surgical population, the most common risk factors for VTE prior to hospitalisation were obesity, pregnancy, longterm immobility and chronic pulmonary disease, whereas chronic heart failure, obesity and chronic pulmonary disease were the commonest in the medical population (Table 4). VTE risk factors in patients during hospitalisation are shown in Table 4.

**Table 4:** Risk factors for thromboembolism among patients on admission

*Risk factor*	*Number (%) of patients*		
	*Surgical (n = 1016)*	*Medical (n = 567)*	*All patients (n = 1583)*
Previous VTE	3 (0.3)	2 (0.4)	5 (0.3)
Chronic pulmonary disease (CPD)	21 (2.0)	45 (8.0)	66 (4.2)
Obesity	104 (10.2)	59 (10.4)	163 (10.3)
Thrombophilia	–	2 (0.4)	2 (0.1)
Long-term immobility	44 (4.3)	41 (7.2)	85 (5.4)
Contraceptive	10 (1.0)	–	10 (0.6)
Varicose veins or venous insufficiency (VVI)	17 (1.7)	7 (1.2)	24 (1.5)
Pregnancy	81 (8.0)	1 (0.2)	82 (5.2)
Chronic heart failure	13 (1.3)	95 (16.8)	108 (6.8)
Post-menopausal hormone replacement therapy	–	1 (0.2)	1 (0.1)
None	764 (75.2)	354 (62.4)	1118 (70.6)

Some patients (surgical and medical) presented with more than one VTE risk factor, and these are shown in Table 5. The most common post-admission risk factors to VTE for both surgical and medical patients were immobilisation with bathroom privileges, complete immobilisation, and admission to intensive care unit (Table 6).

**Table 5:** Patients presenting with more than one risk factor

*Risk factor*	*Number (%) of patients*		
	*Surgical (n = 1016)*	*Medical (n = 567)*	*All patients (n = 1583)*
CPD + obesity	5 (0.5)	1 (0.2)	6 (0.4)
Previous VTE + obesity + VVI + CHF	–	1 (0.2)	1 (0.1)
CPD + long-term immobility	1 (0.1)	-	1 (0.1)
CPD + pregnancy	1 (0.1)	-	1 (0.1)
Obesity + long-term immobility	2 (0.2)	3 (0.5)	5 (0.3)
Obesity + VVI + CHF	1 (0.1)	-	1 (0.1)
Obesity + VVI	4 (0.4)	1 (0.2)	5 (0.3)
Obesity + pregnancy	23 (2.3)	–	23 (1.4)
Long-term immobility + VVI	3 (0.3)	1 (0.2)	4 (0.2)
CPD + obesity + CHF	–	1 (0.1)	1 (0.1)
CPD + long-term immobility + CHF	–	2 (0.3)	2 (0.1)
CPD + VVI + CHF	–	1 (0.1)	1 (0.1)
CPD + CHF	–	10 (1.8)	10 (0.6)
Obesity + CHF	–	5 (0.9)	5 (0.3)
Thrombophilia + long-term immobility + CHF	–	1 (0.1)	1 (0.1)
Long-term immobility + CHF	–	5 (0.9)	5 (0.3)
VVI + CHF	–	1 (0.2)	1 (0.1)

CPD: chronic pulmonary disease, CHF: chronic heart failure, VVI: varicose veins or venous insufficiency.

**Table 6:** Risk factors for thromboembolism among patients during hospitalisation

*Risk factor*	*Number (%) of patients*		
	*Surgical (n = 1016)*	*Medical (n = 567)*	*All patients (n = 1583)*
Admitted to ICU/UCU	195 (19.2)	31 (5.5)	226 (14.5)
Central venous catheter	26 (2.6)	11 (2.0)	37 (2.5)
Mechanical ventilation	106 (10.5)	–	106 (6.7)
Immobile with bathroom privileges	400 (39.5)	280 (49.3)	680 (43.1)
Complete immobilisation	571 (56.3)	132 (23.3)	703 (44.4)
Cancer therapy	20 (1.9)	9 (1.6)	29 (1.8)
Heparin-induced thrombocytopenia	–	–	–

Table 7 shows the contra-indications to pharmacological VTE prophylaxis. Receiving an NSAID on admission and bleeding on admission were the most common contra-indications to pharmacological prophylaxis in surgical patients. In medical patients, significant renal impairment and receiving an NSAID (including aspirin) on admission were the most common contra-indications (Table 7). Of the population at risk for VTE, 112 (25.2%) surgical patients and 115 (32.6%) medical patients were considered to have a high bleeding risk, sufficient to present a contra-indication to anticoagulant prophylaxis.

**Table 7:** Contra-indications to anticoagulant prophylaxis

*Risk factor*	*Number (%) of patients*		
	*Surgical (n = 1016)*	*Medical (n = 567)*	*All patients (n = 1583)*
Admitted to ICU/UCU	195 (19.2)	31 (5.5)	226 (14.5)
Central venous catheter	26 (2.6)	11 (2.0)	37 (2.5)
Mechanical ventilation	106 (10.5)	–	106 (6.7)
Immobile with bathroom privileges	400 (39.5)	280 (49.3)	680 (43.1)
Complete immobilisation	571 (56.3)	132 (23.3)	703 (44.4)
Cancer therapy	20 (1.9)	9 (1.6)	29 (1.8)
Heparin-induced thrombocytopenia	–	–	–

Of patients deemed to be at risk for VTE, 411 (51.5%) received ACCP-recommended types of prophylaxis, of whom 283 (64%) were surgical patients and 128 (36.2%) were medical patients. Anticoagulants were the most frequently used form of VTE prophylaxis in the at-risk population; low-molecular weight heparin was the most commonly prescribed anti-coagulant Table 8. Mechanical prophylaxis (foot pump and graduated compression stockings) were used more frequently in surgical patients than in medical patients.

**Table 8:** ACCP-recommended prophylaxis to VTE received

*Type of prophylaxis*	*Number (%) of patients*		
	*Surgical (n = 1016)*	*Medical (n = 567)*	*All patients (n = 1583)*
Low-molecular weight heparin	506 (49.9)	132 (23.1)	638 (40.2)
Unfractionated heparin	5 (0.5)	11 (2.0)	16 (1.2)
Vitamin K antagonist	7 (0.7)	4 (0.8)	11 (0.8)
Fondaparinux	–	–	–
Other anticoagulants	2 (0.2)	–	2 (0.1)
Foot pump	12 (1.2)	–	12 (2.0)
Graduated compression stockings	54 (5.3)	-	54 (3.4)
Intermittent pneumatic compression	-	-	-

**Fig. 1.**
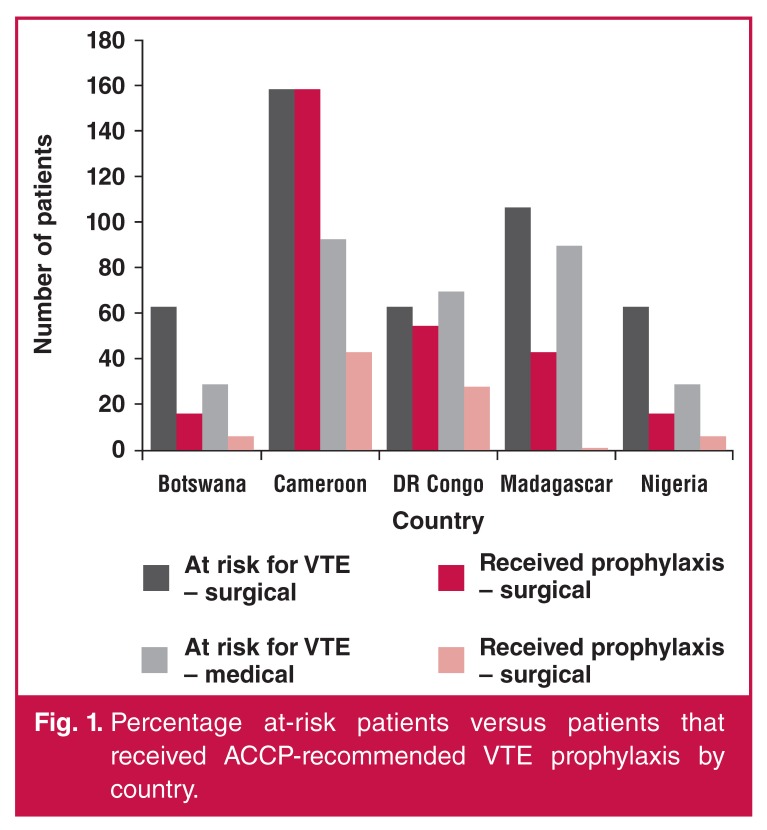
Percentage at-risk patients versus patients that received ACCP-recommended VTE prophylaxis by country.

The proportion of patients at risk for VTE and the use of ACCP-recommended prophylaxis by country are shown in Fig. 1. The proportion of hospital patients at risk for VTE ranged among countries from 23 to 66%, and the proportion of at-risk patients receiving ACCP-recommended prophylaxis ranged from 22 to 80%. The proportion of surgical patients at risk for VTE ranged among countries from 22 to 73%, and for medical patients the range was from 26 to 90%. The use of recommended VTE prophylaxis in surgical patients varied from 25 to 100% between countries, and for medical patients between 1 and 46% (Fig. 1).

## Discussion

The data gathered show a high prevalence of VTE risk among hospitalised patients in sub-Saharan Africa. More than half of all hospitalised patients were at risk for VTE, and medical patients were at higher risk than surgical patients. The data further show that less than half of all at-risk patients received an ACCP-recommended method of prophylaxis.

Data from this survey showed that the proportion of hospital patients at risk for VTE ranged from 23 to 66% and the proportion of at-risk patients receiving ACCP-recommended prophylaxis ranged from 22 to 80%. The proportion of surgical patients at risk for VTE ranged from 22 to 73%, and for medical patients the range was from 26 to 90%. The use of recommended VTE prophylaxis in surgical patients varied from 25 to 100%, and for medical patients between 1 and 46%.

Although there is little information on VTE prevalence and the rate of usage of VTE prophylaxis in Africa,^22^ data from this survey support data from previous studies that have shown a similar trend. Studies conducted elsewhere have reported overall VTE prophylaxis rates ranging from 13 to 64%.

The data showed marked differences between countries in the frequency of use of ACCP-recommended types of prophylaxis, which could be due to many factors, including physician awareness, availability of guidelines, education factors, reimbursement, and national healthcare resources. The use of recommended VTE prophylaxis was particularly poor in medical patients, with prophylaxis received by only 35.1% of medical patients deemed to be at risk. This finding is consistent with other studies that have shown low use of prophylaxis in at-risk medical patients.^12^,^19^

In surgical patients, prophylaxis rates were generally higher. The increased use of prophylaxis in the surgical setting compared with the medical setting could result from several factors. First, the benefits of prophylaxis in the surgical setting have been accepted for many years,^12^ while trials in medical patients have been more recent and physician awareness of VTE risk is lower in this population.^1^ Second, assessment of VTE risk in surgical patients is simpler than in medical patients, the principal criterion being the type of surgery rather than a range of illnesses and risk factors as presented in medical patients.

## Conclusion

While venous thromboembolism is a well-recognised condition in clinical practice in the developed world, with event rates of at least two to three million per year, little attention is paid to this threat in the developing world where the burden of infectious diseases and limited access to healthcare have not recognised VTE as a significant cause of morbidity and mortality. VTE is a major public health issue. It is an easily preventable disease with a substantial risk of morbidity and mortality in patients hospitalised for acute medical and surgical illnesses.

Data from this survey show that in sub-Saharan African, as elsewhere in the world, a large proportion of hospitalised patients are at risk for VTE, and that recommended VTE prophylaxis is underused. Data from this survey are important in providing an overview of the magnitude of VTE prevalence in Africa and the gaps that exist in the prophylaxis of VTE in at-risk patients in hospital settings.
